# Impacts of Embedded Fiber Optic Sensor on Mechanical Properties and Sensing Performances of Intelligent Composites

**DOI:** 10.3390/ma19132713

**Published:** 2026-06-24

**Authors:** Zhe Fan, Rui Bao, Hao Song, Yongwei Tian

**Affiliations:** 1 National Key Laboratory of Strength and Structural Integrity, Institute of Solid Mechanics, School of Aeronautic Science and Engineering, Beihang University, Beijing 100191, China; by1905067@buaa.edu.cn; 2 Changcheng Institution of Metrology & Measurement, Beijing 100095, China; sh208@139.com; 3 State Key Laboratory of Mechanics and Control of Mechanical Structures, Nanjing University of Aeronautics and Astronautics, Nanjing 210016, China; yw_tian@126.com

**Keywords:** fiber Bragg grating, intelligent composites, mechanical property, sensing performance

## Abstract

**Highlights:**

- Up to five FBGs can be embedded without compromising structural integrity.
- Validates FBG sensor feasibility for developing intelligent composites.
- Enables reliable, real-time internal health monitoring of structures.
- Provides reference for optimizing FBG volume fraction and placement.

**Abstract:**

This study presents an experimental and numerical investigation on the impact of embedded fiber optic sensors on the mechanical properties, like tensile, compression, bending and compression-after-impact properties, and sensing performances of intelligent composites. The influence by different volume fractions of embedded fiber optics on the mechanical properties was revealed. Combined with finite element simulations, the effect of embedded sensors on the basic mechanical properties of composite materials was obtained. The sensing performance of the embedded fiber Bragg grating (FBG) sensors was validated through comparison with conventional strain gauges.

## 1. Introduction

Due to their high modulus and high specific strength, composite structures can significantly reduce the structural weight of aircraft [1]. This weight reduction is directly related to the economic and environmental performance of the aviation sector. According to the 2024 Aviation: Benefits Beyond Borders report, the aviation sector and aviation-facilitated tourism supported approximately USD 4.1 trillion in global GDP contribution and 86.5 million jobs in 2023. The direct aviation sector employed approximately 11.6 million people, including about 1.7 million jobs in commercial aerospace manufacturing. Therefore, even moderate improvements in structural weight reduction, durability, and maintenance efficiency can have considerable economic significance for aircraft manufacturing and operation. In this context, the development of intelligent composite structures with embedded sensing capability is important not only for structural performance but also for reducing maintenance costs and improving lifecycle reliability.

Since composites are heterogeneous and anisotropic materials with complex microstructures, nondestructive testing (NDT) of composites is more challenging than that of metallic materials. In the aerospace industry, where composites are extensively used, the current NDT methods for composites suffer from two typical problems. Firstly, most NDT methods are highly localized, making it labor-intensive and time-consuming to detect the damage, due to the large scale of aircraft structures. Secondly, most NDT methods are offline, not only making it unable to provide real-time online detection but also resulting in numerous damages going undetected and unrepaired in a timely manner [2].

Fiber Bragg grating (FBG) sensors are lightweight, flexible and corrosion resistant. This makes them ideal for forming spatially segmented/wavelength-division multiplexed multi-channel monitoring systems by integrating signal sensing and transmission [3]. Embedment of FBG sensors within composite materials enables online monitoring of parameters such as structural stress/strain during both curing process and service life [4], potentially reducing the maintenance costs without heavy burden on structural weight, which is of great significance to enhancing the reliability and intelligence of composite structures. The application potential of FBG sensors in distributed self-diagnostic monitoring networks is widely recognized, making composite structures smarter.

Despite these advantages, embedded FBG sensors also have inherent limitations. First, silica optical fibers are relatively brittle and may be damaged during lay-up, curing, impact, or high-strain loading. Second, the embedded fiber and its coating may disturb the local laminate architecture and introduce resin-rich regions, which can cause local stress concentration. Third, FBG sensors are intrinsically sensitive to both strain and temperature; therefore, temperature compensation is required for accurate strain measurement under variable thermal environments. In addition, an FBG sensor measures local axial strain along the grating region rather than full-field strain, and the interrogation system increases the cost and complexity of the monitoring system. Once embedded and cured inside the laminate, a damaged sensor is also difficult to replace. Therefore, the application of embedded FBG sensors requires a balance between sensing performance, mechanical integrity, durability, and system complexity.

In general, health monitoring of composites using FBG sensors [5,6] primarily in-volves two methods: surface mounting and embedding. Surface-mounted sensors measure surface strain in a manner similar to conventional resistance strain gauges, but exposed fibers are more susceptible to handling damage and environmental effects. Embedded sensors are integrated directly into the laminate [7,8], which enables internal strain and damage monitoring [9,10,11] and can provide a service life consistent with that of the host structure.

Previous studies have demonstrated important progress in embedded-sensor composites, including embedded ultrasonic sensors, multiplexed optical sensors for interfacial fracture monitoring, and bio-inspired embedded vasculature [12,13,14]. Other studies have focused on the strain-transfer behavior of embedded FBG sensors and packaged FBG sensors [15,16,17,18], while the effects of sensor position and embedding method have also been investigated [19,20,21,22]. Nevertheless, most available studies focus on either sensing feasibility, a single loading mode, a specific sensor diameter, or a particular laminate architecture. For example, small-diameter FBG sensors have been successfully used for delamination monitoring in CFRP laminates after low-velocity impact [21], and embedded polymer optical fibers have been reported to have limited influence on the integrity of 3D orthogonal woven composites [17]. However, quantitative comparisons across multiple standardized mechanical properties, different embedded-fiber volume fractions, and different fiber orientations remain insufficient. Therefore, the combined influence of embedded optical-fiber volume fraction, orientation, mechanical integrity, and strain-sensing accuracy still requires systematic clarification.

The scientific novelty of this work is that it combines standardized tensile, compressive, flexural, and compression-after-impact tests with local finite element stress-field analysis and FBG/strain-gauge comparison in the same study. Rather than only demonstrating that FBG sensors can measure strain, this work evaluates the acceptable number and volume fraction of 155 μm polyimide-coated optical fibers that can be embedded while maintaining the mechanical performance of composite laminates. The study also compares 0° and 90° embedding configurations and identifies the local stress-affected zone around the embedded fiber, thereby providing guidance for the design of FBG sensor networks in intelligent composite structures.

Accordingly, this study addresses the following research questions: (1) how does the number/volume fraction of embedded 155 μm optical fibers affect the tensile, compressive, flexural, and compression-after-impact properties of composite laminates? (2) how does the relative orientation between the embedded optical fiber and the reinforcing carbon fibers influence the mechanical response? (3) whether the stress-affected region predicted by the local meso-scale finite element model remains confined near the embedded layer; and (4) whether the embedded FBG sensors can provide strain measurements consistent with conventional resistance strain gauges under tensile and compressive loading. The working assumption is that a limited number of embedded fibers can provide reliable internal strain sensing without causing unacceptable degradation of the host laminate, while an excessive embedded-fiber volume fraction may induce local stress concentration and mechanical degradation.

This paper is organized as follows: Section 2 presents the experimental and numerical investigation of the influence of embedded optical fibers on the mechanical properties of composites. Section 3 evaluates the strain-sensing performance of embedded FBG sensors. Section 4 discusses the implications, limitations, and interpretation of the results. Section 5 concludes the paper.

## 2. Impact on Mechanical Properties

### 2.1. Experiment Preparation

**Figure 1:**
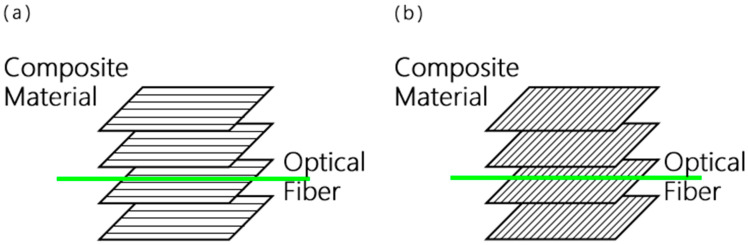
Schematic of optical fiber embedded at: (**a**) 0 degrees; (**b**) 90 degrees.

The composites used in the tests were CCF300/5228 (Guangwei, Weihai, China) and T800/X850 systems (Guangwei, Weihai, China), with a single-ply prepreg thickness of 0.125 mm. A polyimide-coated fiber with a diameter of 155 μm, capable of withstanding temperatures up to 300 °C, was used. The preparation conditions for the tensile, compressive, flexural, and CAI specimens are listed in Table 1. For the 0° specimens, the embedded fiber sensors were aligned parallel to the fibers, while for the 90° specimens they were perpendicular to the fibers, as shown in Figure 1. All specimens underwent ultrasonic C-scan nondestructive testing and were found to be free of significant damage.

The CCF300/5228 composite system(Guangwei, Weihai, China) was used for the tensile, compressive, and CAI specimens, while the T800/X850 composite system was used for the flexural specimens. The finite element model for tensile and compressive analysis was established using the material parameters of the CCF300/5228 system to maintain consistency with the corresponding experimental specimens. The rationale for associating each material system with the corresponding tests has been clarified to avoid over-extrapolation. The CCF300/5228 results are used to discuss tensile, compressive, and CAI responses, whereas the T800/X850 results are used to discuss flexural behavior. Because the two material systems differ in fiber grade and resin formulation, the flexural results are not directly merged with the CCF300/5228 tensile, compressive, or CAI data for quantitative material ranking. Instead, all conclusions concerning the acceptable embedded-fiber volume fraction are based on changes relative to the unembedded baseline within the same material system and same test method.

**Figure 2:**
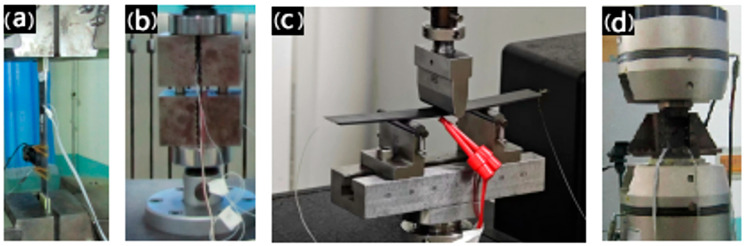
Testing process: (**a**) tensile; (**b**) compressive; (**c**) bending; (**d**) CAI testing processes.

ASTM standard test methods were selected, as shown in Figure 2. Tests on the tensile, compressive, flexural, and compression-after-impact (CAI) properties of composites were conducted both before and after embedding the fiber sensors.

The tensile and compressive stresses were calculated as

(1) $$\sigma =\frac{F}{bt}$$

where *σ* is the tensile or compressive stress, *F* is the applied load, *b* is the specimen width, and *t* is the specimen thickness. The elastic modulus was determined from the slope of the stress-strain curve in the linear elastic region:

(2) $$E=\frac{\Delta \sigma }{\Delta \varepsilon }$$

where *E* is the elastic modulus, △*σ* is the stress increment, and △*ε* is the corresponding strain increment within the linear elastic range.

For the three-point bending test, the flexural strength was calculated as

(3) $$\sigma_{f}=\frac{3F_{\max}L}{2bt^{2}}$$

where *σ_f_* is the flexural strength, *F*_max_ is the maximum bending load, and *L* is the support span. The flexural modulus was calculated as

(4) $$E_{f}=\frac{L^{3}m}{4bt^{3}}$$

where *E_f_* is the flexural modulus and *m* is the slope of the initial linear portion of the load-deflection curve.

The compression-after-impact strength was calculated as

(5) $$\sigma_{CAI}=\frac{F_{\max}}{bt}$$

where *σ_CAI_* is the compression-after-impact strength.

Tensile tests were performed using an INSTRON 8803 machine (Kntest, Jinan, China), with a tensile rate of 2 mm/min. Compression tests were conducted using an HT-9102 computer-controlled servo material testing machine, with a compression rate of 1.3 mm/min. The impact energy was specified as 6.67 J/mm of specimen thickness according to the ASTM D7136/D7137 [26] convention, corresponding to a total impact energy of approximately 13.34 J for the 2 mm thick CAI coupons. The testing process is illustrated in Figure 2.

### 2.2. Experiment Results

All experimental results are presented as mean ± standard deviation. Statistical analyses were performed separately for each test type, fiber orientation, and mechanical property. One-way analysis of variance (ANOVA) was used to evaluate the effect of the number of embedded optical fibers. When the ANOVA result was significant, Tukey’s HSD post hoc test was conducted for pairwise comparisons. A value of *p* < 0.05 was considered statistically significant.

#### 2.2.1. Influences on Tensile Properties

Based on the dimensions of the specimen, the relationship between the number and the volume content of fiber-optic implants into standard test pieces of composites is shown in Table 2. For convenience of description, all the following are expressed in terms of the number of implantations.

**Figure 3:**
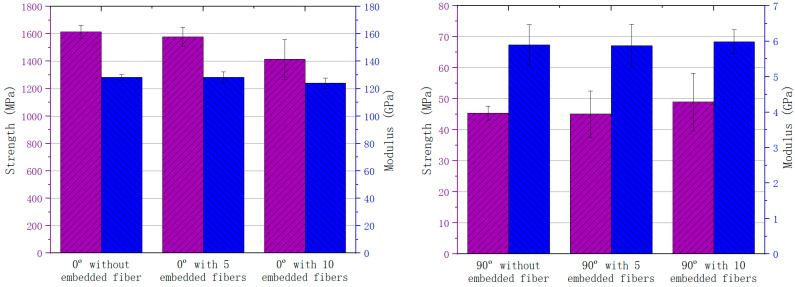
Effect of embedded optical fiber on the tensile mechanical properties of composites.

Tensile tests were conducted on 0-degree and 90-degree specimens with different numbers of embedded fibers. The results of the tensile experiments are shown in Table 3 and Figure 3.

For the 0-degree specimens, in which the optical fibers are parallel to the carbon fibers, both tensile strength and tensile modulus of the composites decrease after embedding the fibers. When five fibers are embedded, the tensile strength decreases by 2.2%, which is slight and acceptable. As the number of embedded fibers increases to 10, the tensile strength decreases by 12.4%, which is much more obvious. Comparatively, the impact on the tensile modulus is smaller. When embedding five fibers, the tensile modulus of the composite remains almost unchanged. When embedding 10 fibers, the tensile modulus decreases by approximately 3.3%. Therefore, for a 0-degree specimen, embedding fewer than five fibers has a minimal effect on the tensile mechanical properties.

For 90-degree specimens, in which the optical fibers are perpendicular to the reinforcing carbon fibers, the influence is different from that in 0-degree specimens. When five fibers are embedded, the tensile strength and modulus remain essentially unchanged; when 10 fibers are embedded, the tensile strength and tensile modulus increase by approximately 7.9% and 1.7%, respectively. This increase should be interpreted cautiously because transverse tensile behavior is mainly governed by the resin matrix, fiber/matrix interface, and local defects rather than by the axial stiffness of the carbon fibers. In the 90-degree configuration, the embedded optical fibers are locally aligned with the loading direction and may act as stiff micro-inclusions that share part of the load, bridge matrix microcracks, and locally suppress crack opening in the resin-rich region. At the same time, the scatter of transverse tensile strength is relatively large; therefore, the observed increase is not considered as a general strengthening effect of FBG embedment but rather as a configuration-dependent response associated with matrix-dominated failure and local crack-bridging effects.

For the 0° tensile strength, one-way ANOVA revealed a significant overall effect of the number of embedded fibers (*p* = 0.009). Post hoc comparisons showed that the 2.2% decrease with five embedded fibers was not statistically significant compared to the unembedded baseline (*p* = 0.32), whereas the 12.4% decrease with 10 embedded fibers was highly significant (*p* = 0.003). For the 0° tensile modulus, no significant differences were found among the three groups (*p* = 0.11). For the 90° tensile strength, the overall ANOVA was not significant (*p* = 0.63), and pairwise comparisons confirmed that neither the slight decrease with five fibers (*p* = 0.89) nor the 7.9% increase with 10 fibers (*p* = 0.14) reached statistical significance. This supports the cautious interpretation that the apparent increase for 90° specimens with 10 fibers may be within experimental scatter.

#### 2.2.2. Influences on the Flexural Properties

**Figure 4:**
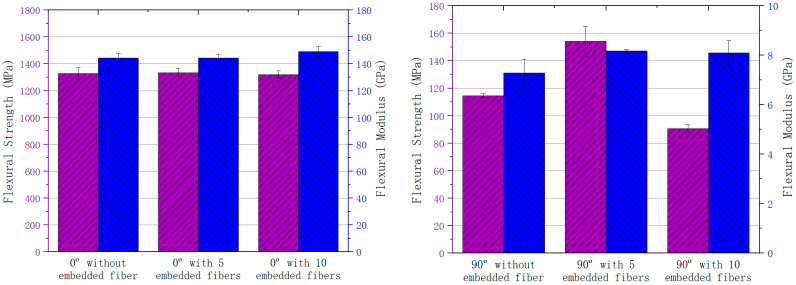
Effect of embedded optical fiber on the flexural mechanical properties of composites.

The flexural performance for 0-degree and 90-degree specimens are shown in Table 4, and the trends in average strength and modulus are illustrated in Figure 4.

For 0-degree specimens, variations of flexural strength are within 1% among zero-fiber cases, five-fiber cases and 10-fiber cases, indicating that the influence of fiber embedment can be neglected. In contrast, the difference in flexural modulus between the zero-fiber cases and five-fiber cases can also be neglected. However, the flexural modulus increases by 3.3% in the 10-fiber cases compared to the unembedded specimens. Therefore, it can therefore be concluded that there is no harmful influence for fiber embedment on flexural properties in 0-degree specimens.

For 90-degree specimens, the flexural strength of the five-fiber specimens increased by 34.7%, and the flexural modulus increases by 12.1%, which are significant differences compared to unembedded specimens. However, as the number of embedded fibers increases to 10, the flexural strength decreases severely by 21.0%, while the flexural modulus increases by 11.1%.

For 0° flexural strength and modulus, one-way ANOVA indicated no statistically significant differences among the three groups (strength: *p* = 0.82; modulus: *p* = 0.07). The slight increases and decreases observed are all within experimental scatter. For 90° flexural properties, the overall ANOVA was highly significant for both strength (*p* < 0.001) and modulus (*p* = 0.004). Post hoc comparisons revealed that the 34.7% increase in strength with five embedded fibers was significant (*p* < 0.001), while the 21.0% decrease with 10 fibers was also significant (*p* < 0.001) compared to the unembedded baseline. The five-fiber and 10-fiber groups also differed significantly from each other (*p* < 0.001). For the 90° flexural modulus, the 12.1% increase with five fibers was significant (*p* = 0.004), but the 11.1% increase with 10 fibers, although still higher than the baseline, was not significantly different from the five-fiber group (*p* = 0.79). This suggests that the modulus enhancement occurs already at five fibers and does not further increase with more fibers.

#### 2.2.3. Influences on Compressive Properties

**Figure 5:**
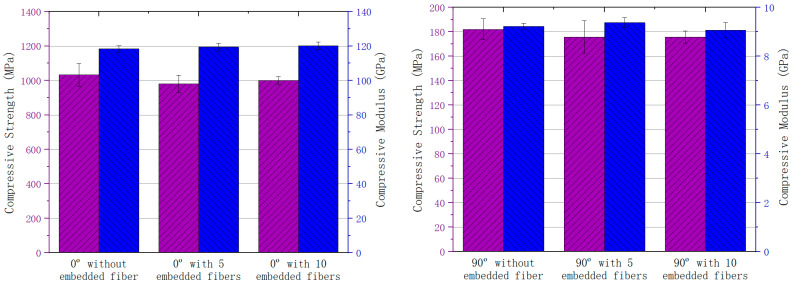
Effect of embedded optical fiber on the compressive mechanical properties of composites.

The compressive performance data for 0-degree composites with no embedded fibers and with five embedded fibers are shown in Table 5. The trends in compressive strength and modulus are illustrated in Figure 5.

For 0-degree specimens, it can be observed that the average compressive strength of 0-degree composite specimens with five embedded fibers is around 980 MPa, which is approximately 5% lower than that of specimens without embedded fibers. After embedding 10 fibers, the compressive strength slightly improves, being about 3% lower than that of the unembedded specimens. The change in compressive modulus of the composite remains within 2% after embedding fibers. Therefore, for 0-degree layered composites, embedding fibers slightly decreases the compressive strength while the compressive modulus remains essentially unchanged.

For 90-degree specimens, the compressive performance data for 90-degree composites with no embedded fibers and with five and 10 embedded fibers are shown in the table below, and the trends in compressive strength and modulus are illustrated in the figure. It can be seen that for 90-degree composites, the changes in compressive strength and modulus after embedding fibers are within 3%. This indicates that the impact of this fiber volume content on the compressive performance of 90-degree composites is negligible.

For 0° compressive strength, one-way ANOVA gave *p* = 0.15, indicating no statistically significant overall difference among the three groups. Although the average strength with five fibers was 5.1% lower than the baseline, the pairwise comparison was not significant (*p* = 0.11). The 3.1% reduction with 10 fibers was also not significant (*p* = 0.26). For 0° compressive modulus, all groups were similar (*p* = 0.42). For 90° compressive strength, the overall ANOVA was not significant (*p* = 0.48), and none of the pairwise differences reached significance (five fibers vs. baseline: *p* = 0.30; 10 fibers vs. baseline: *p* = 0.30). Similarly, for 90° compressive modulus, no significant differences were detected (*p* = 0.10). These results confirm that the influence of up to 10 embedded fibers on compressive properties is statistically negligible for both fiber orientations.

#### 2.2.4. Influences on CAI Properties

The influence of embedded fibers on the CAI performance of composites is shown in Table 6. Embedding 12 fibers increases the average indentation depth by about 2.8%, increases the length of the back surface cracks by about 33.3%, and reduces the post-impact compressive strength by around 5.1%. Therefore, embedding fibers slightly decreases the composites’ CAI performance.

The reduction in CAI strength and the increase in back-surface crack length may be related to local stress concentration and crack initiation around the embedded optical fibers [27,28]. From a multiscale perspective, the embedded optical fiber, coating layer, resin-rich region, and the surrounding carbon-fiber/epoxy laminate form a heterogeneous local region. The mismatch in stiffness and geometry among these constituents can alter the local stress field during impact and subsequent compression. Nanomechanical studies have shown that local mechanical heterogeneity can strongly affect damage initiation, especially in regions where local stiffness, interface bonding, and matrix deformation are nonuniform. For CFRP composites, crack initiation at the nanoscale can occur through competing mechanisms such as fiber/matrix interface debonding and in-matrix crack initiation. Therefore, the observed increase in back-surface crack length after embedding optical fibers may be associated with the coalescence of microcracks initiated around resin-rich or interface-sensitive regions. This suggests that both bulk mechanical properties and local nanoscale damage mechanisms should be considered when designing embedded FBG sensor networks in composite laminates.

For the CAI strength, the 5.1% reduction observed with 12 embedded fibers was statistically significant (independent *t*-test, *p* = 0.047). The indentation depth increase (2.8%) was not significant (*p* = 0.32), while the back-surface crack length increase (33.3%) was significant (*p* = 0.03). This indicates that although the CAI strength degradation is modest, the change in crack morphology is more pronounced and statistically reliable.

### 2.3. Simulation

For composite laminates containing optical fibers, a finite element local meso-model was established to analyze the impact on tensile and flexural properties. This model identifies how the optical fibers influence the composite’s mechanical properties and analyzes the effect of different embedding layers on the composite’s mechanical performance.

The finite element models were generated using structured three-dimensional solid elements with mesh refinement around the optical fiber and resin-rich region. The element type, total element number, minimum element size near the optical fiber, and convergence criterion were Abaqus/Standard C3D8R elements, approximately 450,000 elements, 0.005 mm, and the standard force residual tolerance. Mesh convergence was assessed by comparing the apparent stiffness and the size of the stress-affected region after further mesh refinement; the solution was considered mesh-independent when the variation was below 2%. The simulation results were compared with the experimental trends at the qualitative level: the stress-affected zone remained local, which is consistent with the small changes in most measured mechanical properties for limited embedded-fiber volume fractions.

#### 2.3.1. Tensile Performance Analysis

**Figure 6:**
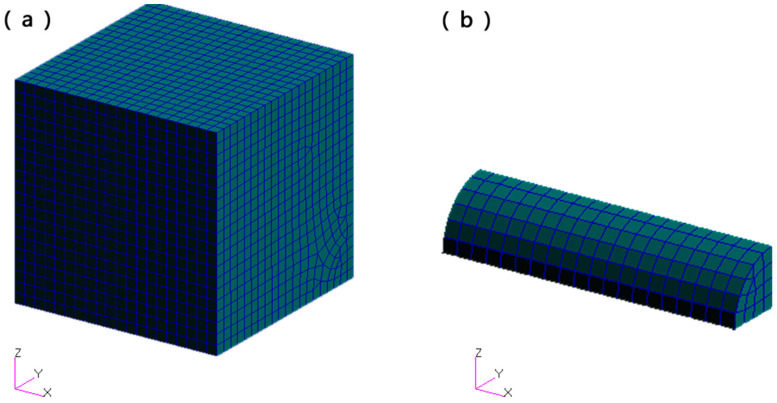
(**a**) Finite element local meso-model; (**b**) finite element local meso-model of optical fiber part.

Taking the 0-degree specimen 1# as an example, a local meso-model was established to analyze the mechanical properties. The specimen parameters are as follows: 155 μm diameter optical fiber + one optical fiber + 0-degree layer + middle layer. The selected meso-model has dimensions of 0.5 mm in length, width, and thickness, forming a cube containing 1/4 of the optical fiber. The CCF300/5228 system and the optical fiber were considered as two separate parts. After establishing the solid model, mesh discretization was performed, as shown in Figure 6a,b.

Since the selected unit cell contains 1/4 of the optical fiber, symmetric boundary conditions were applied. Displacement in the X direction was constrained to zero on one exterior surface perpendicular to the *X*-axis (fiber direction). Similarly, the displacement in the Y direction was constrained to zero on one exterior surface perpendicular to the *Y*-axis, and the displacement in the Z direction was constrained to zero on one exterior surface perpendicular to the *Z*-axis. After applying the boundary conditions to the meso-model, a 2% displacement constraint of the model size was applied to a surface perpendicular to the *X*-axis for the static analysis.

**Figure 7:**
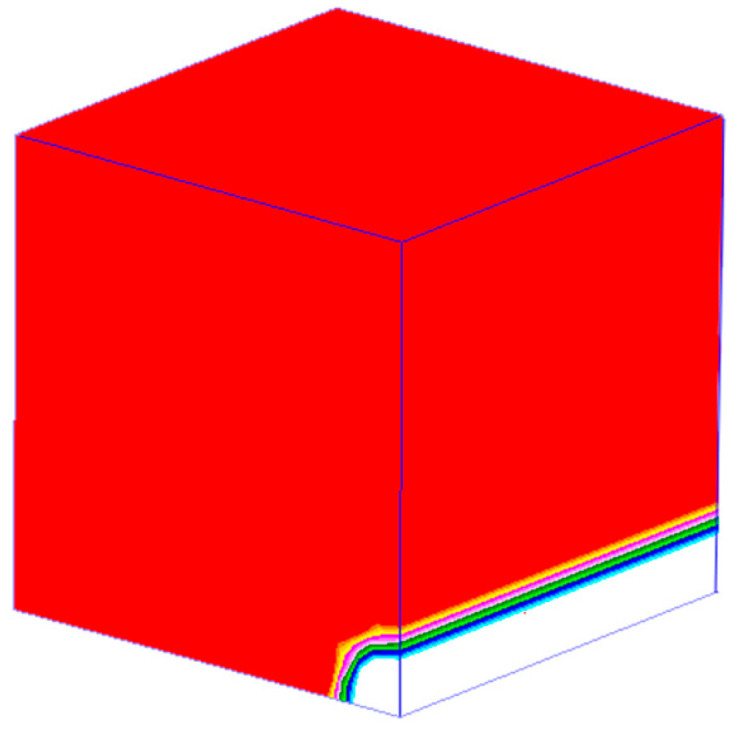
Tensile stress contour (including 155 μm optical fiber).

The elastic modulus and other parameters of the CCF300/5228 system and the optical fiber are detailed in Table 7. Figure 7 shows the stress cloud of the material after stretching, and it can demonstrate the stress distribution at the optical fiber. Away from the optical fiber, the composite material properties are not affected, so as to obtain the region of the optical fiber’s influence on the material properties, which is a circular region with a diameter of about 200 μm, i.e., only 22.5 μm outside the diameter of the optical fiber range of stress concentration occurs, and the scope of the influence is small.

**Figure 8:**
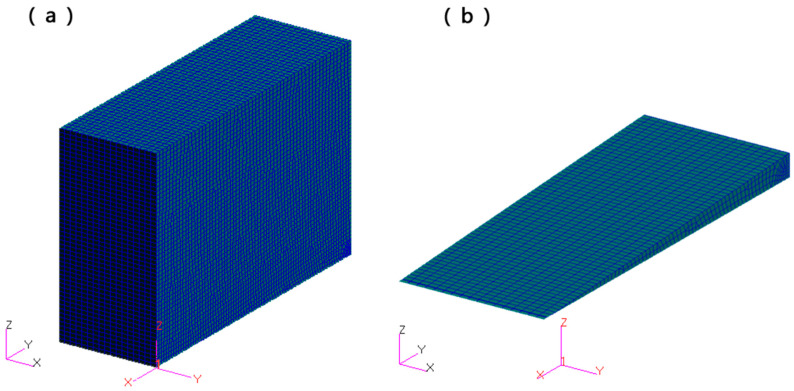
Finite element local meso-model of: (**a**) cubic unit; (**b**) optical fiber part.

Microscopic observations of Section 1 reveal that embedding optical fibers in 90-degree materials produces spindle-shaped resin-rich areas. A local meso-model was established to analyze the mechanical properties of the 90-degree specimen. The specimen parameters are as follows: 155 μm diameter optical fiber + one optical fiber + 0-degree layer + middle layer. The selected meso-model has dimensions of 0.5 mm in length, 1.5 mm in width, and 1 mm in thickness, forming a cube containing 1/4 of the optical fiber. The resin-rich area has an arcuate middle section with straight ends. The final finite element model is shown in Figure 8.

Since the selected unit cell contains 1/4 of the optical fiber, symmetric boundary conditions were applied. The displacement in the X direction was constrained to zero on one exterior surface perpendicular to the *X*-axis (fiber direction). Similarly, the displacement in the Y direction was constrained to zero on one exterior surface perpendicular to the *Y*-axis, and the displacement in the Z direction was constrained to zero on one exterior surface perpendicular to the *Z*-axis. After applying the boundary conditions to the meso-model, a displacement constraint of 2% of the model size was applied to another surface perpendicular to the *X*-axis for static analysis.

**Figure 9:**
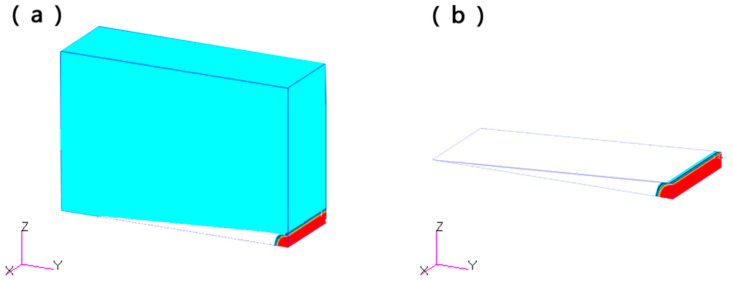
Tensile stress contour (including 155 μm optical fiber) for fiber and resin-rich area. (**a**) specimen; (**b**) optical fiber part.

Figure 9 demonstrates the stress contour of the material under tensile load. It can be observed that the stress distribution around the optical fiber is distinct. The performance of the composite material remains unaffected in regions far from the optical fiber, indicating that the impact area of the optical fiber on the material’s performance is an approximately diamond-shaped region with a length of 2.5 mm and a width of 200 μm.

The optical fiber is embedded between two layers of composite material, with a single-ply prepreg thickness of 125 μm. Therefore, the thickness of the fiber’s impact area is less than the thickness of the two composite layers. This means that the influence of the embedded fiber on the tensile properties of the composite material does not exceed the embedded layer’s range. Since the overall impact of the embedded fiber on the tensile properties of the composite material is minimal, and the fiber’s position does not significantly affect the overall tensile properties, it can be concluded that different embedding layers of the optical fiber do not significantly impact the overall tensile properties of the composite material.

#### 2.3.2. Flexural Performance Analysis

##### 0-Degree Materials

**Figure 10:**
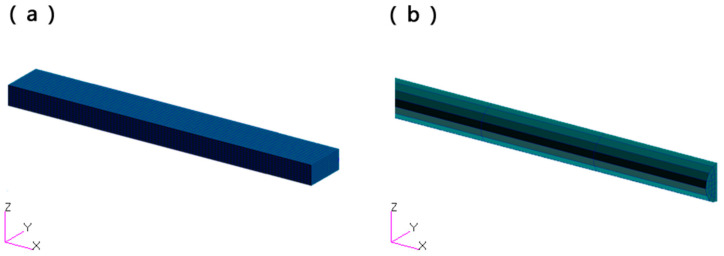
Finite element local meso-model for flexural performance analysis of: (**a**) specimen; (**b**) optical fiber part.

Taking the 0-degree specimen as an example, 1/4 of the specimen was used to establish the finite element model for flexural performance analysis. The specimen parameters are as follows: 155 μm diameter optical fiber + one optical fiber + 0-degree layer + middle layer. The selected meso-model has dimensions of 48 mm in length, 6.5 mm in width, and 1.5 mm in thickness. The finite element model is shown in Figure 10.

On one exterior surface perpendicular to the *X*-axis (fiber direction), the displacement in the X direction was constrained to zero. Similarly, the displacement in the Y direction was constrained to zero on one exterior surface perpendicular to the *Y*-axis. On the bottom surface perpendicular to the *Z*-axis, the displacement in the Z direction was constrained at *X* = −48 mm. After applying these boundary conditions to the meso-model, a displacement constraint in the Z direction was applied to the top surface at *X* = 0 mm for static analysis. This setup allows for evaluating the flexural performance of the composite material with the embedded optical fiber.

**Figure 11:**
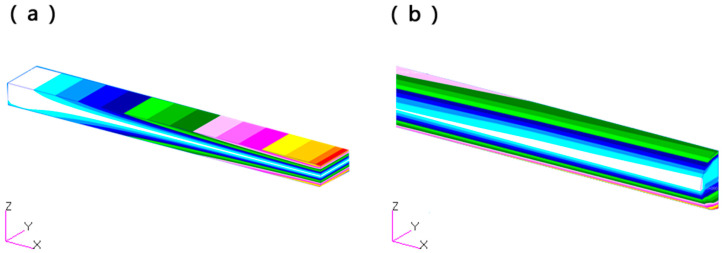
Flexural stress contour of the composite containing a 155 μm optical fiber under the applied load: (**a**) overall view; (**b**) enlarged view around the optical fiber.

Figure 11 shows the flexural stress contour of the material after applying the load. The stress distribution around the optical fiber is clearly visible. The performance of the composite material remains unaffected in regions far from the optical fiber, indicating that the fiber’s impact area is a circular region with a diameter of approximately 160 μm.

##### 90-Degree Materials

On one exterior surface perpendicular to the *X*-axis (fiber direction), the displacement in the X direction was constrained to zero. Similarly, the displacement in the Y direction was constrained to zero on one exterior surface perpendicular to the *Y*-axis. On the bottom surface perpendicular to the *Z*-axis, the displacement in the Z direction was constrained at *X* = −48 mm. After applying these boundary conditions to the meso-model, a displacement constraint in the Z direction was applied to the top surface at *X* = 0 mm for static analysis.

**Figure 12:**
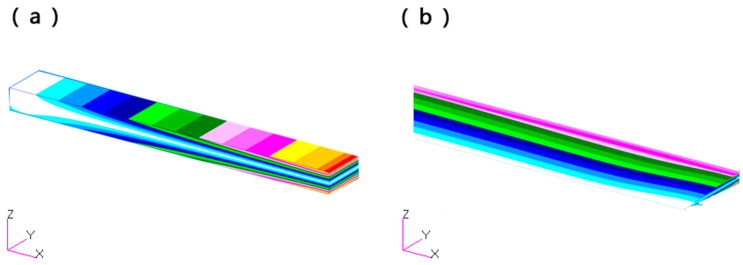
Flexural stress contour of the composite containing a 155 μm optical fiber after bending deformation : (**a**) overall view; (**b**) enlarged view around the optical fiber.

Figure 12 indicates the flexural stress contour of the material after bending. It can be observed that the stress distribution around the optical fiber is distinct. The performance of the composite material remains unaffected in regions far from the optical fiber, indicating that the fiber’s impact area is an approximately diamond-shaped region with a length of 2.5 mm and a width of 160 μm.

The optical fiber is embedded between two layers of composite material, with a single-ply prepreg thickness of 125 μm. Therefore, the thickness of the fiber’s impact area is less than that of the two composite layers combined. This means that the influence of the embedded fiber on the flexural properties of the composite material does not exceed the embedded layer’s range. The position of the embedded fiber has some impact on the overall flexural performance, with the influence being smaller when the fiber is closer to the center of the material and larger when closer to the surface. However, since the overall impact of the embedded fiber on the composite’s flexural performance is minimal, it can be concluded that different embedding layers of the optical fiber do not significantly impact the composite material’s overall flexural performance.

The predicted stress-affected zone sizes are small compared to the overall specimen dimensions. For example, in 0° tension, the influence region is a circle of approximately 200 μm diameter (only about 22.5 μm beyond the fiber surface), and, in 90° tension, it is a diamond-shaped region of 2.5 mm × 200 μm. These limited local disturbances are consistent with the experimental observation that embedding five fibers (volume fraction ≤ 0.63%) reduces most mechanical properties by less than 5%. When the number of embedded fibers increases to 10, the cumulative disturbed volume grows, which corresponds to the more evident degradation seen in 0° tensile strength (−12.4%) and 90° flexural strength (−21.0%). Thus, the local meso-scale model provides a plausible explanation for the volume-fraction-dependent trends measured experimentally.

### 2.4. Summary

The impact of embedding different numbers of optical fiber sensors on the mechanical properties of composites is summarized in Table 8. Taking the composite without embedded sensors as the baseline, the symbols indicate the level of impact as follows: ☆ indicates a slight increase compared to the unembedded specimen, ★ indicates a decrease within 5%, ★★ indicates a decrease between 5% and 10%, and ★★★ indicates a decrease greater than 10%.

From the table, it can be concluded that:

1. Compared with modulus, strength is more sensitive to the embedment of optical fiber sensors.
2. Except the 0° tensile specimens and 90° flexural specimens with 10 embedded optical fibers, the impact of embedded optical fibers on the strength and modulus of composites is less than 5%.
3. Impacts of embedding five optical fibers with a diameter of 155 µm on variations of mechanical properties are within the range of 5%.

## 3. Impact on Sensing Performance

### 3.1. Strain Monitoring During Tensile Testing

The embedded FBG sensors had a central Bragg wavelength of 1550 nm, a grating length of 10 mm, and a nominal strain sensitivity of 1.20 pm/με. The sensors were supplied by Wuhan Ligong Optical Technology Co., Ltd. (Wuhan, China). The reflected wavelength was recorded using a Wuhan Ligong Optical FBG interrogator (Wuhan Ligong Optical Technology Co., Ltd., Wuhan, China) with a sampling rate of 1 kHz.

**Figure 13:**
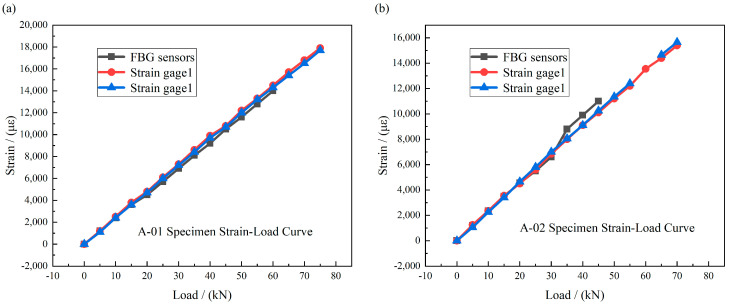

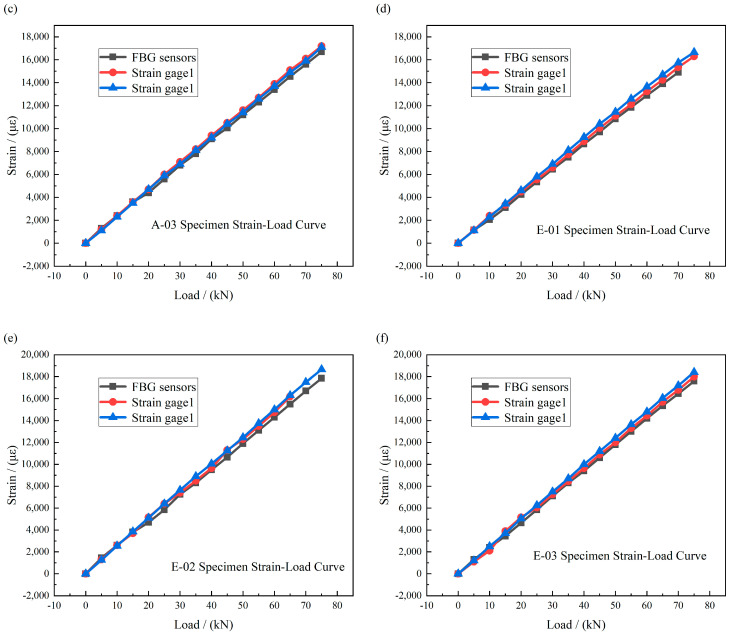
Comparison of strain measurements from strain gauges and fiber Bragg gratings embedded between layers 9–10: (**a**) A-01 specimen strain-load curve; (**b**) A-02 specimen strain-load curve; (**c**) A-03 specimen strain-load curve; (**d**) E-01 specimen strain-load curve; (**e**) E-02 specimen strain-load curve; (**f**) E-03 specimen strain-load curve.

During the tensile test, the strain signals measured by FBGs embedded in different layers and by surface strain gauges are shown in Figure 13. It can be seen that there is a good linear relationship between the strain monitored by the FBG sensors and the externally applied load. Therefore, FBG sensors embedded within the composite can monitor the internal strain changes of the material in real-time manner, and the strain values measured by the FBG sensors are consistent with those measured by the strain gauges. Additionally, strain data obtained from the embedded FBG sensors are slightly lower than strain gauge measurements. This discrepancy may be due to the polyimide resin protective layer around the fiber absorbing a small amount of energy during the tensile process as the strain in the composite increases.

Specifically, when the applied load exceeds 30 kN, the strain values from the FBG sensors become greater than those from the strain gauges. As the load increases to approximately 50 kN, the FBG signal disappears. This signal loss is likely associated with severe local strain concentration, optical-fiber fracture, local debonding, delamination, or matrix cracking near the embedded FBG region. However, because no post-test optical microscopy, SEM, or X-ray CT inspection was performed for the failed sensor location, this interpretation is considered a plausible explanation rather than direct evidence of the exact failure mechanism.

Overall, the trend of the experimental results measured by the FBG sensors and strain gauges matches well. The FBG sensors embedded between the composite layers can accurately and effectively measure the internal strain changes of the composite laminates, with strain measurements exceeding 10,000 με.

### 3.2. Strain Monitoring During Compression Testing

**Figure 14:**
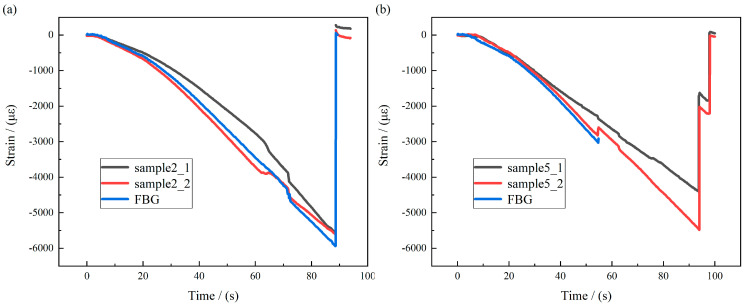
Comparison of strain data measured by embedded FBG sensors and surface strain gauges under compression conditions: (**a**) specimen 2; (**b**) specimen 5.

Strain monitoring was conducted during compression testing for specimens with 0-degree plies. Strain gauges were affixed to both sides of the specimens, and FBGs were embedded in the middle layer. The strain measured by the strain gauges and the fiber sensors is shown in Figure 14.

Figure 14 shows that for the 0-degree compression specimen with a single embedded sensor, the strain measured by the FBG sensor is consistent with the trend observed from the resistance strain gauges. However, due to uneven force distribution during compression, measurements from the two strain gauges differ from those of the FBG sensor. During the compression of Specimen 2, the measurements from the embedded FBG sensor were between those of the two surface strain gauges. In the compression process of Specimen 5, the FBG sensor signal disappeared after −3000 µε, accompanied by the sound of fiber breakage, indicating that cracking occurred near the sensor. Overall, the embedded fiber sensors can effectively monitor the compressive performance of the composite material.

## 4. Discussion

Although the present work focuses on standardized mechanical testing, strain monitoring, and finite element analysis, further microscopic characterization is still required to directly reveal the fracture morphology around embedded optical fibers. The present work did not evaluate sensing performance during flexural or CAI loading because these tests introduce strong strain gradients, local damage evolution, and, for impact, high-rate transient events that require different sensor layouts and high-speed interrogation. In future work, scanning electron microscopy, high-resolution X-ray computed tomography, and digital image correlation will be combined to visualize surface fracture morphology, internal delamination, resin-rich regions, and local crack initiation near embedded sensors. Such multiscale characterization will provide more direct evidence for correlating local damage mechanisms with macroscopic mechanical degradation.

Several limitations should be noted. First, the number of specimens in each group is limited, and the statistical analysis should therefore be interpreted together with the experimental scatter. Second, two different carbon/epoxy material systems were used for different test types; therefore, comparisons are made within each material system and test method rather than by directly ranking all mechanical results together. Third, the finite element analysis is a local meso-scale model and is not a fully validated coupon-scale predictive model. Fourth, post-test microscopy or X-ray CT was not performed for all failed FBG locations, so the exact sensor failure mechanism remains to be directly confirmed. These limitations define the scope of the present conclusions and motivate the future work described below.

Future work will focus on five aspects. First, the volume fraction, spacing, and embedding path of FBG sensors should be further optimized to balance sensing coverage and mechanical integrity. Second, coating and interfacial design should be investigated to reduce local stress concentration and improve strain transfer between the composite laminate and the embedded sensor. Third, temperature compensation and multi-parameter calibration should be incorporated to improve sensing accuracy under complex service environments. Fourth, long-term durability tests, including fatigue loading, repeated impact, hygrothermal aging, and thermal cycling, should be conducted to evaluate the reliability of embedded FBG sensors during service. Finally, multiscale characterization methods such as SEM, high-resolution X-ray computed tomography, and digital image correlation should be combined with finite element modeling to clarify the relationship between local damage mechanisms and macroscopic mechanical performance. These future studies will support the development of more reliable intelligent composite structures for aerospace applications.

## 5. Conclusions

In this paper, FBG sensors with different numbers of embedded optical fibers were integrated into composite laminates, and the influence of sensor implantation on the fundamental mechanical properties of the composites was systematically investigated through theoretical analysis and experimental validation. The strain-sensing performance of the embedded FBG sensors was also compared with that of conventional resistance strain gauges. The results indicate that the tensile strength of the composites is more sensitive to FBG embedment than the elastic modulus, and the influence becomes more pronounced as the fiber volume fraction increases. For composites embedded with five optical fibers, the reduction in strength is less than 5% compared with specimens without embedded sensors. In addition, the embedded FBG sensors are capable of accurately and reliably measuring the internal strain of composite laminates during both tensile and compressive loading processes. The results further show that the embedding position of the FBG sensors has no significant effect on the strain measurement outcomes.

## Figures and Tables

**Table 1:** Specimen preparation conditions.

Test Type	Test Standard	Fiber Orientation	Specimen Dimensions	Number of Embedded Fibers
Tensile	ASTM D3039 [23]	0 Degree	250 mm × 15 mm × 1 mm	0, 5, 10
		90 Degree	175 mm × 25 mm × 2 mm	0, 5, 10
Flexural	ASTM D7264 [24]	0 Degree	150 mm × 13 mm × 3 mm	0, 5, 10
		90 Degree	150 mm × 13 mm × 3 mm	0, 5, 10
Compressive	ASTM D6641 [25]	0 Degree	140 mm × 12 mm × 2 mm	0, 5, 10
		90 Degree	140 mm × 12 mm × 2 mm	0, 5, 10
CAI	ASTM D7137 [26]	[45/0/-45/90]4s	140 mm × 12 mm × 2 mm	0, 12

**Table 2:** Embedded volume content versus number of fibers.

	Tensile	Compressive	Bending	CAI
0	0%	0%	0%	0%
1	0.130%	0.080%	0.050%	0.005%
5	0.630%	0.390%	0.240%	/
10	1.260%	0.790%	0.480%	/
12	/	/	/	0.057%

**Table 3:** Tensile properties data of composites with embedded fiber sensors (0°, 90°).

Specimen ID		Strength (MPa)	Modulus (GPa)	Specimen ID		Strength (MPa)	Modulus (GPa)
0° without embedded fiber	1-3	1581.88	126.73	90° without embedded fiber	4-1	47.26	5.57
	1-5	1697.30	129.11		4-2	45.60	5.28
	1-6	1582.61	125.88		4-3	43.25	6.69
	1-7	1599.90	130.20		4-4	47.92	6.25
	1-8	1607.43	129.55		4-5	42.95	5.68
	Average	1613.82	128.29		Average	45.40	5.89
	Standard Deviation	47.95	1.88		Standard Deviation	2.26	0.57
0° with 5 embedded fibers	2-1	1604.45	129.17	90° with 5 embedded fibers	5-1	39.97	6.31
	2-2	1628.41	126.63		5-2	38.13	4.82
	2-3	1580.70	133.13		5-3	48.80	6.13
	2-4	1457.05	122.36		5-4	56.22	6.00
	2-5	1619.78	130.11		5-5	42.43	6.10
	Average	1578.08	128.28		Average	45.11	5.87
	Standard Deviation	70.04	4.04		Standard Deviation	7.41	0.60
0° with 10 embedded fibers	3-1	1477.57	125.17	90° with 10 embedded fibers	6-1	56.12	6.22
	3-3	1181.62	123.57		6-4	42.62	5.96
	3-4	1369.61	126.57		6-5	55.90	5.57
	3-5	1536.83	117.95		6-6	35.69	6.40
	3-6	1502.47	127.13		6-7	54.69	5.79
	Average	1413.62	124.08		Average	49.00	5.99
	Standard Deviation	143.98	3.69		Standard Deviation	9.33	0.33

**Table 4:** Flexural properties data of composites with embedded fiber sensors (0°, 90°).

Specimen Type	Specimen ID	Flexural Strength (MPa)	Flexural Modulus (GPa)	Specimen Type	Specimen ID	Flexural Strength (MPa)	Flexural Modulus (GPa)
0° without fibers	1-1	1367.94	140.79	90° without fibers	1-1	116.44	7.28
	1-2	1365.19	141.01		1-2	115.38	7.80
	1-3	1348.96	146.38		1-3	114.74	7.72
	1-4	1284.85	145.79		1-4	113.51	6.41
	1-5	1274.08	147.75		1-5	112.53	7.24
	Average	1328.20	144.34		Average	114.52	7.29
	Standard Deviation	45.24	3.22		Standard Deviation	1.54	0.55
0° with 5 fibers	2-1	1344.23	141.56	90° with 5 fibers	2-1	167.86	8.20
	2-2	1292.94	147.89		2-2	151.33	8.16
	2-3	1353.85	142.59		2-3	155.66	8.24
	2-4	1315.85	142.75		2-4	158.44	8.17
	2-5	1363.44	146.72		2-5	138.29	8.08
	Average	1334.06	144.30		Average	154.31	8.17
	Standard Deviation	29.07	2.81		Standard Deviation	10.82	0.06
0° with 10 fibers	2-2	1307.20	146.98	90° with 10 fibers	2-2	92.77	7.77
	2-5	1315.98	146.03		2-5	94.41	7.92
	2-6	1307.04	146.01		2-6	88.31	7.55
	2-7	1306.86	153.78		2-7	87.75	8.71
	2-8	1367.20	152.87		2-8	89.16	8.54
	Average	1320.86	149.13		Average	90.48	8.10
	Standard Deviation	26.20	3.86		Standard Deviation	2.94	0.50

**Table 5:** Compressive properties data of 0-degree composites with and without embedded fibers.

Type	Specimen ID	Compressive Strength (MPa)	Compressive Modulus (GPa)	Type	Specimen ID	Compressive Strength (MPa)	Compressive Modulus (GPa)
0° without embedded fiber	1-1	1085.09	120.43	90° without embedded fiber	1-1	193.87	9.25
	1-2	993.18	120.18		1-2	178.89	8.99
	1-3	1117.22	117.40		1-3	175.56	9.34
	1-4	1006.56	117.56		1-4	173.90	9.24
	1-6	959.65	116.19		1-6	187.83	9.27
	Average	1032.34	118.35		Average	182.01	9.22
	Standard Deviation	66.09	1.86		Standard Deviation	8.54	0.13
0° with 5 embedded fibers	3-3	922.57	116.70	90° with 5 embedded fibers	3-3	191.13	9.34
	3-4	1005.54	117.39		3-4	160.09	9.29
	3-5	994.76	122.08		3-5	185.17	9.72
	3-6	1042.20	121.46		3-6	164.44	9.31
	3-7	935.10	119.25		3-7	177.84	9.20
	Average	980.03	119.38		Average	175.73	9.37
	Standard Deviation	50.13	2.39		Standard Deviation	13.26	0.20
0° with 10 embedded fibers	4-1	980.15	119.68	90° with 10 embedded fibers	4-1	178.26	8.99
	4-3	1027.20	122.32		4-3	176.75	9.53
	4-4	1015.54	121.87		4-4	166.88	8.86
	4-5	996.76	119.67		4-5	178.73	8.75
	4-7	980.41	116.71		4-7	178.05	9.19
	Average	1000.01	120.05		Average	175.73	9.06
	Standard Deviation	21.03	2.23		Standard Deviation	5.003652	0.31

**Table 6:** CAI test data.

Specimen Type	Statistic	Indentation Depth (mm)	Back Surface Crack Length (mm)	Impact Load (kN)	CAI Strength (MPa)
Without embedded fibers	Average	3.28	79.29	64.23	218.5
Without embedded fibers	Standard deviation	0.56	17.69	14.89	12.3
With 12 embedded fibers	Average	3.37	105.69	62.86	207.4
With 12 embedded fibers	Standard deviation	0.40	28.47	11.24	11.8

**Table 7:** Basic material properties.

Property	CCF300/5228	Optical Fiber
Longitudinal Tensile Modulus (GPa)	126	70
Transverse Tensile Modulus (GPa)	9.24	70
Poisson’s Ratio	0.313	0.17
Longitudinal Shear Modulus (GPa)	4.43	29.9

**Table 8:** Impact of embedded optical fibers on the mechanical properties of composites.

		Tensile		Modulus	
Number of Embedded Fibers		5 Fibers	10 Fibers	5 Fibers	10 Fibers
Tensile Test Material System (CCF300/5228)	0°	★	★★★	★	☆
	90°	★	☆	☆	★
Flexural Test Material System (T800/X850)	0°	☆	☆	☆	★
	90°	☆	★★★	☆	☆
Compressive Test Material System (CCF300/5228)	0°	★	★	☆	☆
	90°	★	★	☆	★
CAI Test Material System (CCF300/5228)	Isotropic	/	★ (12 Fibers)	/	/

## Data Availability

The original contributions presented in this study are included in the article. Further inquiries can be directed to the corresponding author.
